# Genome-wide association study of coronary artery disease among individuals with diabetes: the UK Biobank

**DOI:** 10.1007/s00125-018-4686-z

**Published:** 2018-07-12

**Authors:** Tove Fall, Stefan Gustafsson, Marju Orho-Melander, Erik Ingelsson

**Affiliations:** 1 Department of Medical Sciences, Molecular Epidemiology, Uppsala University, EpiHubben, MTC-huset, 751 85 Uppsala, Sweden; 2 Science for Life Laboratory, Uppsala University, Uppsala, Sweden; 3 Department of Clinical Sciences in Malmö, Lund University, Malmö, Sweden; 4 Department of Medicine, Division of Cardiovascular Medicine, Stanford University of Medicine, Stanford, CA USA; 5 Stanford Cardiovascular Institute, Stanford University of Medicine, Stanford, CA USA

**Keywords:** Diabetes, Genetic epidemiology, Ischaemic heart disease, UK Biobank

## Abstract

**Aims/hypothesis:**

Coronary artery disease (CAD) is a common complication among individuals with diabetes. A better understanding of the genetic background of CAD in this population has the potential to suggest novel molecular targets for screening, risk assessment and drug development.

**Methods:**

We performed a genome-wide association study of CAD in 15,666 unrelated individuals (3,968 CAD cases and 11,698 controls) of white British ancestry with diabetes at inclusion in the UK Biobank study. Our results were compared with results from participants without diabetes.

**Results:**

We found genome-wide significant evidence for association with CAD at the previously well-established *LPA* locus (lead variant: rs74617384; OR 1.38 [95% CI 1.26, 1.51], *p* = 3.2 × 10^−12^) and at 9p21 (lead variant: rs10811652; OR 1.19 [95% CI 1.13, 1.26], *p* = 6.0 × 10^−11^). Moreover, other variants previously associated with CAD showed similar effects in the participants with and without diabetes, indicating that the genetic architecture of CAD is largely the same.

**Conclusions/interpretation:**

Our results indicate large similarities between the genetic architecture of CAD in participants with and without diabetes. Larger studies are needed to establish whether there are important diabetes-specific CAD loci.

**Electronic supplementary material:**

The online version of this article (10.1007/s00125-018-4686-z) contains peer-reviewed but unedited supplementary material, which is available to authorised users.

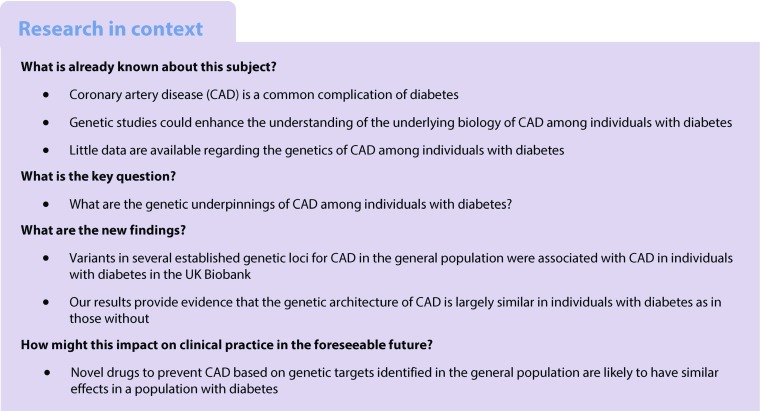



## Introduction

Diabetes mellitus confers about two-fold increased risk of coronary artery disease (CAD) [1]. Better understanding of the genetic background of CAD among individuals with diabetes can suggest novel molecular targets for screening, risk assessment, and drug development. The aim of our study was to identify genetic variation associated with CAD among individuals with diabetes and to assess previously established CAD loci.

## Methods

### Study sample

We used an algorithm [2] to define diabetes status among UK Biobank participants at baseline (2006-2010) based on self-reported disease, medication and age at diabetes onset. Participants were genotyped on the Affymetrix UK Biobank Lung Exome Evaluation (UK BiLEVE) Axiom array or the Affymetrix UK Biobank Axiom array. Quality control and imputation using the Haplotype Reference Consortium (HRC) was conducted centrally at the UK Biobank, yielding a total of about 39 million single-nucleotide polymorphisms (SNPs) [3]. Out of 24,680 individuals with available genetic data and possible/probable type 1 or type 2 diabetes, we proceeded with 20,644 individuals deemed unrelated with genetic data that passed quality control [3]. Of these, 4,978 individuals of non-white non-British ancestry were excluded from main analyses but were eligible for secondary analyses where we defined (1) ‘European, non-British white ancestry’, (2) ‘Asian or Asian British ancestry’ and (3) ‘Black or Black British ancestry’ subsets based on self-reported ancestry and being within ±2 SD of the two first principal components of that population (ESM Fig. 1). Hence, the main data set contained 15,666 individuals of white British ancestry. The outcome of interest was CAD based on UK Biobank’s baseline assessment verbal health interview, combined with linked data from hospital admissions and death registries (accessed 28 November 2017; last event recorded 15 January 2016; ESM Methods). We considered CAD events before and after diabetes onset. UK Biobank received ethics approval from the National Health Service Research Ethics Service (reference 11/NW/0382) and participants gave informed consent. The current analyses were conducted under approved UK Biobank data applications 11140 and 13721.

### Genome-wide association study

Using the Genetic Association Study Power Calculator (http://csg.sph.umich.edu/abecasis/cats/gas_power_calculator/reference.html, accessed 14 February 2018), we found that for a significance level of 5 × 10^−8^ we had >80% power to detect variants with an allele frequency of 25% and a per-allele OR ≥1.17 and to detect variants with an allele frequency of 1% and an OR ≥1.80 (ESM Fig. 2). We tested 9,087,334 autosomal variants with a quality score (IMPUTE2 information metric) >0.8 and a minor allele count ≥30 in cases and controls in a series of logistic regression models adjusting for age, sex, the first 20 principal components and genotyping batch (3 levels; the UK BiLEVE, UK Biobank release 1 and 2). The association tests were performed in PLINK v2.00 [4] (www.cog-genomics.org/plink/2.0/)) using dosages (additive coding). We applied distance-based pruning followed by conditional analysis as described in the ESM Methods. Gene-based and gene-set pathway analyses with MAGMA v1.6 [5] implemented in FUMA [6] (http://fuma.ctglab.nl/, accessed 5 March 2018) were performed based on SNP association results, as described in the ESM Methods. Regional association plots were created with the Locuszoom tool [7].

### Secondary analyses

In secondary analyses, we assessed (1) associations of identified SNPs with incident CAD (comparing 2,316 individuals with CAD diagnosis after diabetes diagnosis with controls); (2) associations of identified loci in other ancestry groups; (3) 63 variants previously reported as being associated with CAD in the Coronary ARtery DIsease Genome wide Replication and Meta-analysis plus The Coronary Artery Disease Genetics (CARDIoGRAMplusC4D) consortium [8] using a Bonferroni-corrected *p* value (0.05/61 available SNPs = 0.0008) in the 15,666 individuals with diabetes, as well as in 321,281 non-related individuals of British ancestry without diabetes from the UK Biobank; (4) two variants previously reported as being associated with CAD among individuals with diabetes: *GLUL* rs10911021 [9] and *PPARG* rs1801282 (Pro12Ala) [10]; (5) associations of identified SNPs among those with probable type 1 and type 2 diabetes only and among those with probable type 2 diabetes only; and (6) associations of identified SNPs excluding individuals with angina only.

## Results

### Genome-wide association study

**Fig. 1:**
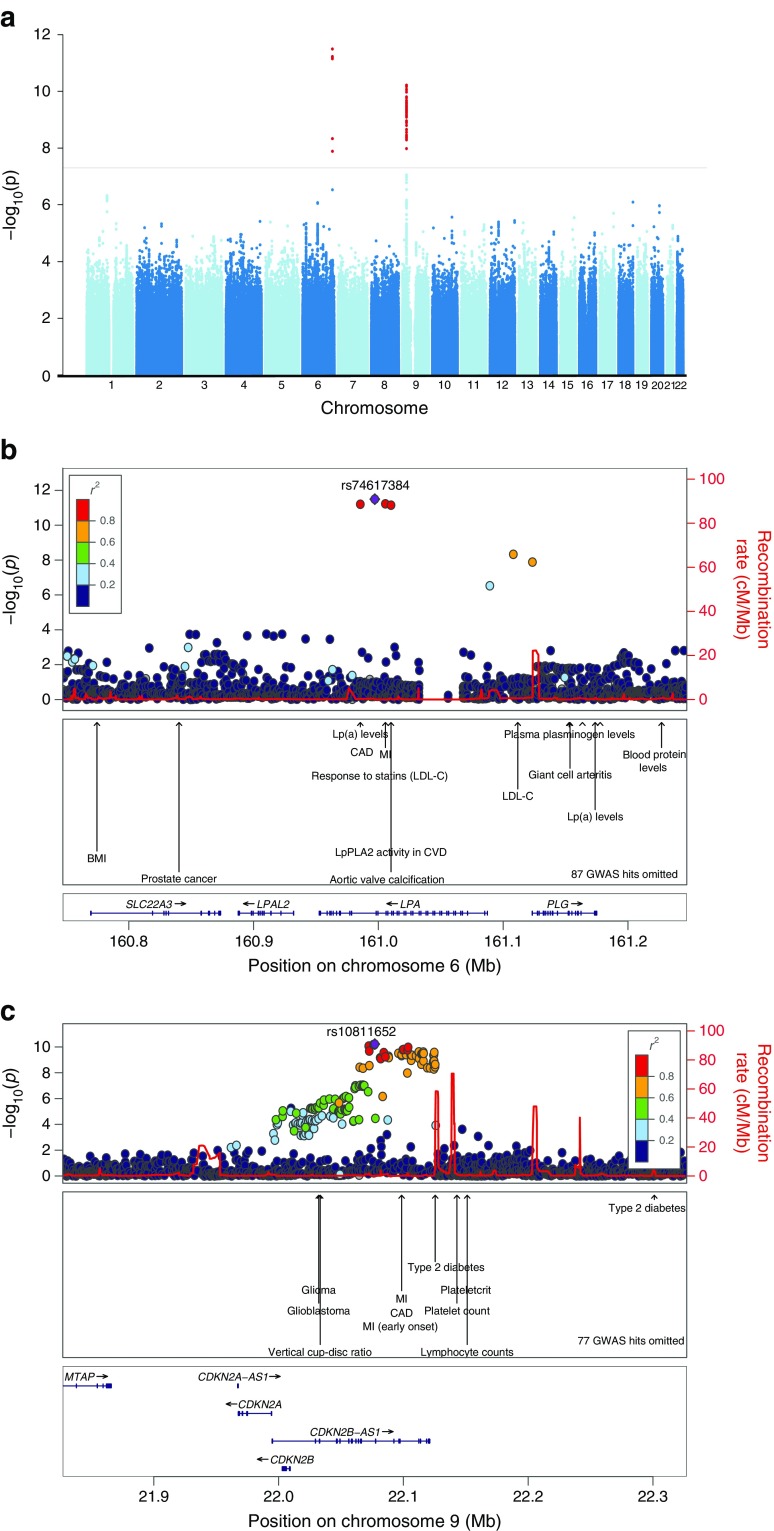
Overview of the association results from the genome-wide association study of CAD in UK Biobank participants of white British ancestry and with diabetes mellitus (3968 CAD cases and 11,698 controls). Each tested SNP is visualised as a dot with location on the genome shown on the *x*-axis and –log_10_-transformed *p* values on the *y*-axis. (**a**) Results for all chromosomes with the consensus threshold for genome-wide significance (5 × 10^−8^) marked with a horizontal line. (**b**, **c**) Regional (Locuszoom [7]) plots of the two loci *LPA* (**b**) and 9p21 (**c**). SNPs are coloured based on their correlation with the index SNP in each locus in the regional plots. Previously reported phenotypes in genome-wide association studies are annotated. *CDKN2A-AS1* is also known as *CDKN2A-DT*. CVD, cardiovascular disease; LDL-C, LDL-cholesterol; Lp(a), lipoprotein(a); LpPLA_2_, lipoprotein-associated phospholipase A_2_; MI, myocardial infarction

In our analyses of 15,666 individuals with diabetes (3,968 CAD cases and 11,698 controls; ESM Table 1, ESM Table 2), we found genome-wide significant evidence for association with CAD at two loci (Fig. 1a–c). The strongest association was observed in the *LPA* locus at 6q25 (lead variant: rs74617384) with the T allele (frequency 8.2%) having an OR of 1.38 (95% CI 1.26, 1.51, *p* = 3.2 × 10^−12^). The second association was found in 9p21 (lead variant: rs10811652), with the C allele (frequency 49.4%) having an OR of 1.19 (95% CI 1.13, 1.26, *p* = 6.0 × 10^−11^). No additional independent variants were identified in these loci. No indication of genomic inflation was observed (ESM Fig. 3). Results from gene-based analyses suggested variation in *PSRC1* and *CYGB*-*PRCD* to be associated with CAD **(**ESM Fig. 4**)**. The *CYGB*-*PRCD* locus has not been reported associated with CAD previously, but we noted one variant, rs72860151, showing suggestive association (*p* = 3.5 × 10^−5^) in summary statistics from CARDIoGRAMplusC4D [8]. No gene sets were significantly enriched in the pathway analysis.

### Secondary analyses

**Fig. 2:**
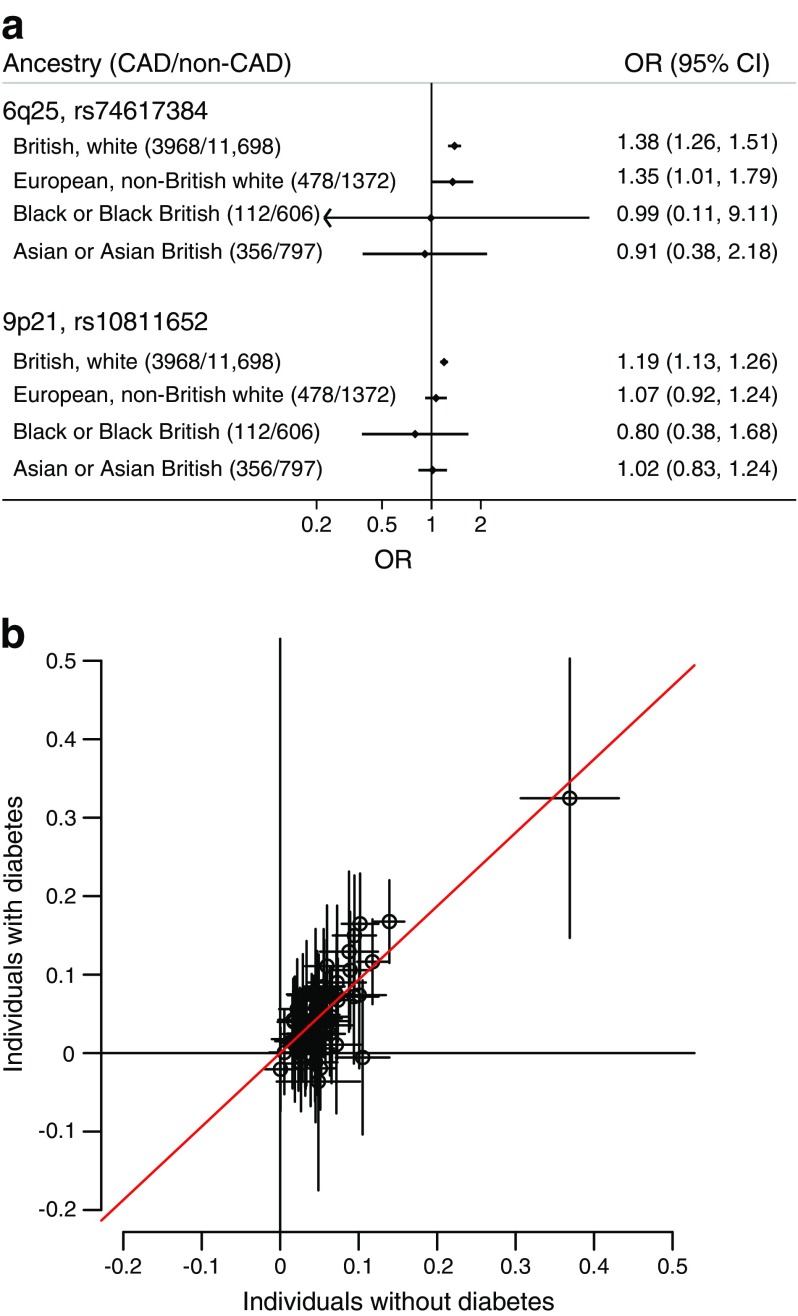
(**a**) Association of the two genetic variants associated with CAD among individuals with diabetes in different ancestry groups. (**b**) Scatterplot of the estimated CAD effects for 61 previously reported CAD variants in UK Biobank participants with diabetes (*n* = 15,666) compared with the UK Biobank participants without diabetes (*n* = 321,281). Estimates on the scatterplot are log_*e*_-transformed ORs with 95% CIs. The slope from a weighted linear regression without intercept is 0.93 (95% CI 0.80, 1.07) and is shown as a red line

In our secondary analyses including only 2,316 CAD cases occurring after diabetes onset, the two top variants had similar effect estimates: rs74617384, OR 1.35 (95% CI 1.20, 1.50, *p* = 1.6 × 10^−7^) and rs10811652, OR 1.17 (95% CI 1.10, 1.25, *p* = 1.5 × 10^−6^). We found evidence of an association (OR 1.35; 95% CI 1.01, 1.79) of rs74617384 with CAD in the European, non-British population, but not in the other ethnic groups (Fig. 2a). Five of 61 previously reported CAD variants were associated with CAD in the population with diabetes at a *p* value of <0.0008 (ESM Table 3). In addition to variants in the *LPA* and 9p21 loci, variants in the *SORT1* and *KCNE2* loci were also associated. Out of the remaining 56 variants, 13 were associated at a *p* value of <0.05 and 36 additional variants showed the same effect direction and similar effect sizes as in the non-diabetic population (Fig. 2b; *p* value from binomial test <10^−6^). The previously reported association of two variants with CAD in individuals with diabetes could not be replicated for either variant in the present study. The effect estimates were OR 1.01 (0.95, 1.07) for each T allele of rs10911021; and 1.05 (0.97, 1.14) for each G allele of Pro12Ala (rs1801282), respectively. Association results were similar using different definitions of diabetes and different definitions of CAD (ESM Table 4).

## Discussion

We report the largest genome-wide association study of CAD among individuals with diabetes to date. There are three main findings. First, we report two genome-wide significant loci (*LPA* and 9p21) previously linked to CAD in the general population with similar effect sizes. Second, among 61 previously identified CAD variants in the general population, two—*SORT1* and *KCNE2*—were significantly associated with CAD among individuals with diabetes. Moreover, in the diabetic population, 49 of the remaining 56 non-significant variants showed effect directions and sizes consistent with those observed in the non-diabetic population. Third, we could not replicate findings from previous studies on diabetes-specific CAD loci, highlighting the limitations of small study samples, lack of replication and/or candidate gene-based approaches. Overall, our study provides evidence that the genetic mechanisms underlying CAD in participants with diabetes are similar to those in individuals without diabetes.

Our gene-based analyses provided some evidence for association of variation in or near three genes with CAD in the population with diabetes: *PSRC1*, *CYGB* and *PRCD. PSRC1* lays within the *CELSR2–PSRC1–SORT1* locus, which has been associated with LDL-cholesterol and CAD, where there is convincing evidence for *SORT1* being the causal gene [11]. *CYGB* and *PRCD* are located in the same locus on chromosome 17, where we also found some orthogonal evidence of a signal in the CARDIoGRAMplusC4D data. The gene *CYGB* encodes the protein cytoglobin, which is a recently discovered globin expressed in fibroblasts and smooth muscle cells, and is reported to be an important regulator of cardiovascular tone in mice [12]. The results of these gene-based analyses need to be replicated in other data sets, but tentatively suggest *CYGB* as an interesting candidate gene, especially given increasing recognition of the important role of smooth muscle cells in atherosclerosis [13].

In this study, we did not detect genetic variants specific to CAD in the population with diabetes. In our study, power was limited for lower allele frequencies and smaller effect sizes, and so we cannot exclude the possibility that such variants will be detected in larger future studies. However, the general interpretation of our findings is that the genetic architecture of CAD in the population without diabetes and in those with diabetes is largely similar.

Limitations of this study include a limited power in other ethnicities than white British, although it is reasonable to assume that the underlying biology, but not necessarily linkage disequilibrium structure, is similar across ethnic backgrounds. Another limitation is that individuals in the control group are likely to have subclinical or clinical CAD not captured by our definition. Such misclassification likely attenuated our estimates and thereby increases the chance of false negative findings and underestimation of effects. Finally, our study included only participants with previously diagnosed diabetes and a mean age of 63 years, which is rather young for a diabetes cohort. In the future, when the biomarker data (including glucose and HbA_1c_) become available in the UK Biobank, it will be possible to extend the current study to those with undiagnosed diabetes.

Our results indicate large similarities between the genetic architecture of CAD in the individuals with and without diabetes. Larger studies are needed to establish whether there are diabetes-specific CAD loci with smaller effect sizes.

## Electronic supplementary material

ESM (PDF 360 kb)

## Data Availability

This research has been conducted using the UK Biobank resource. All bona fide researchers can apply to use the UK Biobank resource for health-related research that is in the public interest (www.ukbiobank.ac.uk/register-apply/). CARDIoGRAMplusC4D data was accessed from www.cardiogramplusc4d.org/.

## Abbreviations

BiLEVE: Biobank Lung Exome Evaluation
CAD: Coronary artery disease
CARDIoGRAMplusC4D: Coronary ARtery DIsease Genome wide Replication and Meta-analysis plus The Coronary Artery Disease Genetics
SNP: Single-nucleotide polymorphism
