# Supplementary material for: Genome-wide association study of coronary artery disease among individuals with diabetes: the UK Biobank
Source: Diabetologia. 2018 Jul 12;61(10):2174–9. doi: 10.1007/s00125-018-4686-z (PMC6133153; doi:10.1007/s00125-018-4686-z)
Supplement: Supplementary file 1 — (PDF 360 kb) [file 125_2018_4686_MOESM1_ESM.pdf]

Electronic Supplementary Material

Genome-wide association study of coronary artery disease among individuals with diabetes: The UK Biobank

Fall *et al.*

ESM Methods

Outcome definition

CAD was defined as having a recorded death or hospitalization with primary or secondary diagnosis recorded with the International Statistical Classification of Diseases and Related Health Problems (ICD) version 10 codes I20, angina pectoris; I21, acute myocardial infarction; 122 subsequent ST elevation (STEMI) and non-ST elevation (NSTEMI) myocardial infarction; I23, certain current complications following ST elevation (STEMI) and non-ST elevation (NSTEMI) myocardial infarction; I24, other acute ischaemic heart diseases; or I25, chronic ischaemic heart disease. We further considered the following ICD-9 codes: 410, acute myocardial infarction; 411, other acute and subacute forms of ischaemic heart disease; 412, old myocardial infarction or 413 angina pectoris. We also considered those individuals as CAD that had the following surgical intervention recorded: K40, saphenous vein graft replacement of coronary artery; K41, other autograft replacement of coronary artery; K42, allograft replacement of coronary artery; K43 prosthetic replacement of coronary artery; K44, other replacement of coronary artery; K45, connection of thoracic artery to coronary artery; K46, other bypass of coronary artery; K49, transluminal balloon angioplasty of coronary artery; K50, other therapeutic transluminal operations on coronary artery; K75, percutaneous transluminal balloon angioplasty and insertion of stent into coronary artery. Further, individuals were classified as CAD if they reported angina pectoris or myocardial infarction at the verbal interview. If the participant was uncertain of the type of illness they had had, then they described it to the interviewer (a trained nurse) who attempted to place it within the coding tree. If the illness could not be located in the coding tree, the interviewer entered a free-text description of it. These free-text descriptions were subsequently examined by a doctor and, where possible, matched to entries in the coding tree. Free-text descriptions which could not be matched with very high probability have been marked as "unclassifiable". Individuals not fulfilling the above criteria were defined as not having CAD.

Pruning and conditional analysis

We identified regions containing one or more SNPs with $p<5e-8$ (“index SNPs”) by screening a window of 500kb adjacent to the first index SNP on each chromosome. If no additional SNPs were identified, the region was limited to that specific SNP, and screening was continued at the next index SNP. If additional index SNPs were present in this 500kb window, the window was expanded with 300kb from the last SNP, and we screened for additional SNPs with $p<5e-8$, until there were no more SNPs with $p<5e-8$ within the next 300kb. From this pruning, a number of regions was identified containing one to several index SNPs. Within each region, the SNP with lowest p-value was assigned as the lead SNP. For each region, association analysis was repeated including all index SNPs in the region as well as the lead SNP from other regions at the same chromosome, and any SNP with $p<5e-8$ in this analysis was considered as an independent locus.

Gene-based analysis and pathway analysis

We applied gene-based and gene-set pathway analyses with MAGMA v1.6[2] implemented in FUMA[3], based on the SNP association results from our GWAS study. In the gene-based analysis, SNP association data were aggregated to the level of 18,205 protein coding genes. An F-test was used to compute the gene p-value based on a multiple linear principal components regression model.[2] The gene p-values and a computed gene correlation matrix were then used to perform gene-set analysis. Gene sets were obtained from the “C2 collection: Curated gene sets” and Goterms from the Molecular Signatures Database (MSigDB) v5.2[4] and a total of 10,894 gene sets were tested. For both gene-based and gene-set pathway analyses, Bonferroni-corrected p-value thresholds were used to denote significance.

ESM Table 1. Descriptive statistics of the 19,387 individuals with diabetes at baseline in the UK Biobank included in this study.

Ancestry	Variable	Individuals with coronary artery disease	Individuals without coronary artery disease
**British, white**	N	3,968	11,698
	Age at visit	62.7 (5.6)	60.2 (7.0)
	Age at DM diagnosis	52.4 (12.2)	51.2 (12.6)
	Body mass index	32.1 (5.6)	31.4 (5.9)
	Systolic blood pressure	143 (20)	144 (18)
	Diastolic blood pressure	79 (11)	82 (10)
	Male	2,936 (74.0%)	7,037 (60.2%)
	Type 1 diabetes	268 (6.8%)	945 (8.1%)
	Insulin treatment	1,020 (25.9%)	2,396 (20.6%)
**Europe, non-British white**	N	478	1,372
	Age at visit	61.3 (6.9)	59.3 (7.2)
	Age at DM diagnosis	51.3 (12.9)	50.8 (12.0)
	Body mass index	32.7 (6.0)	31.6 (6.0)
	Systolic blood pressure	143 (19)	142 (18)
	Diastolic blood pressure	80 (11)	82 (10)
	Male	351 (73.4%)	796 (58.0%)
	Type 1 diabetes	38 (7.9%)	111 (8.1%)
	Insulin treatment	118 (24.9%)	287 (21.2%)
**Black or Black British**	N	112	606
	Age at visit	61.1 (7.1)	56.9 (8.1)
	Age at DM diagnosis	50.7 (12.2)	48.0 (11.0)
	Body mass index	32.2 (6.4)	31.5 (6.0)
	Systolic blood pressure	146 (23)	144 (18)
	Diastolic blood pressure	83 (11)	84 (11)
	Male	50 (44.6%)	305 (50.3%)
	Type 1 diabetes	5 (4.5%)	16 (2.6%)
	Insulin treatment	32 (29.4%)	135 (22.6%)
**Asian or Asian British**	N	356	797
	Age at visit	60.3 (7.4)	57.0 (7.8)
	Age at DM diagnosis	47.4 (12.3)	46.9 (12.7)
	Body mass index	29.2 (5.1)	28.6 (5.0)
	Systolic blood pressure	141 (20)	141 (18)
	Diastolic blood pressure	79 (12)	82 (10)
	Male	286 (80.3%)	472 (59.2%)
	Type 1 diabetes	14 (3.9%)	16 (2.0%)
	Insulin treatment	87 (24.9%)	119 (15.5%)

ESM Table 2. Source of data for definition of coronary artery disease in the main analysis of 15,666 individuals of white British ancestry with 3,968 coronary artery disease events. Each person is counted only once. For explanations of ICD-codes, see ESM Methods.

Source	Definition	n	%
**Death register**	ICD10, I21-I25	252	6.4
**Hospital register**	ICD10, I21-I25; ICD9, 410-412	3041	76.6
**Verbal interview**	"Myocardial infarction"	122	3.1
**Surgical interventions**	K40-K46, K49, K50, K75	2	0.05
**Hospital register**	ICD10, I20; ICD9, 413	342	8.6
**Verbal interview**	"Angina Pectoris"	209	5.3

ESM Table 3. Previously reported variants from the CARDIoGRAMplusC4D genetic consortium for CAD in the general population compared to association results in the UK Biobank diabetes population (n=15,666) and the UK Biobank population without diabetes at baseline (n=321,281)

Genetic variant	Chr	Position, hg19	Candidate gene	Effect allele	Effect allele freq.	CARDIoGRAM	UK Biobank [diabetes]	UK Biobank [without diabetes]
rs3798220	6	160961137	*SLC22A3-LPAL2-LPA**	C	0.02	1.42 (1.26, 1.60)	1.38 (1.16, 1.65)	1.45 (1.36, 1.54)
rs4977574	9	22098574	*9p21*	G	0.49	1.21 (1.19, 1.23)	1.18 (1.12, 1.25)	1.15 (1.13, 1.17)
rs3217992	9	22003223	*9p21*	T	0.37	1.14 (1.12, 1.16)	1.12 (1.06, 1.19)	1.12 (1.10, 1.15)
rs6725887	2	203745885	*WDR12*	C	0.12	1.14 (1.11, 1.18)	1.08 (1.00, 1.16)	1.07 (1.04, 1.11)
rs3918226	7	150690176	*NOS3*	T	0.08	1.14 (1.09, 1.19)	0.99 (0.90, 1.10)	1.11 (1.07, 1.15)
rs17114036	1	56962821	*PPAP2B*	A	0.91	1.13 (1.09, 1.17)	1.08 (0.98, 1.18)	1.11 (1.07, 1.14)
rs9982601	21	35599128	*KCNE2 (gene desert)*	T	0.13	1.12 (1.08, 1.15)	1.16 (1.08, 1.25)	1.10 (1.07, 1.13)
rs646776	1	109818530	*SORT1*	T	0.78	1.11 (1.08, 1.13)	1.18 (1.11, 1.26)	1.11 (1.08, 1.13)
rs12526453	6	12927544	*PHACTR1*	C	0.67	1.10 (1.08, 1.12)	1.07 (1.01, 1.13)	1.05 (1.03, 1.07)
rs8042271	15	89574218	*MFGE8-ABHD2*	G	0.96	1.10 (1.06, 1.14)	0.96 (0.84, 1.11)	1.05 (1.00, 1.11)
rs16986953	2	19942473	*AK097927*	A	0.07	1.09 (1.06, 1.12)	1.14 (1.03, 1.26)	1.09 (1.05, 1.13)
rs445925	19	45415640	*APOE-APOC1*	G	0.89	1.09 (1.05, 1.13)	1.07 (0.99, 1.17)	1.10 (1.06, 1.13)
rs501120	10	44753867	*CXCL12*	T	0.87	1.08 (1.06, 1.11)	1.08 (1.00, 1.17)	1.05 (1.02, 1.08)
rs17465637	1	222823529	*MIA3*	C	0.71	1.08 (1.06, 1.10)	1.05 (0.99, 1.11)	1.07 (1.05, 1.09)
rs7173743	15	79141784	*ADAMTS7*	T	0.54	1.08 (1.06, 1.10)	1.07 (1.02, 1.13)	1.06 (1.04, 1.08)
rs11206510	1	55496039	*PCSK9*	T	0.81	1.08 (1.05, 1.11)	1.00 (0.93, 1.07)	1.04 (1.01, 1.07)
rs12413409	10	104719096	*CYP17A1-CNNM2-NT5C2*	G	0.92	1.08 (1.05, 1.11)	1.09 (0.99, 1.21)	1.08 (1.04, 1.12)
rs11556924	7	129663496	*ZC3HC1*	C	0.62	1.08 (1.05, 1.10)	1.00 (0.95, 1.06)	1.05 (1.03, 1.07)
rs579459	9	136154168	*ABO*	C	0.21	1.08 (1.05, 1.10)	1.01 (0.95, 1.08)	1.03 (1.01, 1.06)
rs1122608	19	11163601	*LDLR*	G	0.75	1.08 (1.05, 1.10)	1.02 (0.96, 1.08)	1.04 (1.02, 1.06)

rs7692387	4	156635309	*GUCY1A3*	G	0.82	1.07 (1.05, 1.10)	1.08 (1.01, 1.16)	1.06 (1.03, 1.08)
rs9515203	13	111049623	*COL4A1/A2*	T	0.73	1.07 (1.05, 1.10)	1.02 (0.97, 1.09)	1.03 (1.00, 1.05)
rs56062135	15	67455630	*SMAD3*	C	0.77	1.07 (1.05, 1.10)	1.04 (0.98, 1.11)	1.04 (1.02, 1.06)
rs1412444	10	91002927	*LIPA*	T	0.34	1.07 (1.05, 1.09)	1.04 (0.99, 1.10)	1.04 (1.02, 1.06)
rs2075650	19	45395619	*APOE-APOC1*	G	0.14	1.07 (1.04, 1.11)	1.11 (1.03, 1.20)	1.09 (1.06, 1.12)
rs9818870	3	138122122	*MRAS*	T	0.17	1.07 (1.04, 1.10)	1.04 (0.97, 1.12)	1.07 (1.04, 1.09)
rs515135	2	21286057	*APOB*	C	0.82	1.07 (1.04, 1.10)	1.08 (1.01, 1.15)	1.03 (1.01, 1.06)
rs3184504	12	111884608	*SH2B3*	T	0.49	1.07 (1.04, 1.09)	1.02 (0.97, 1.08)	1.07 (1.05, 1.09)
rs974819	11	103660567	*PDGFD*	T	0.29	1.07 (1.04, 1.09)	1.05 (0.99, 1.11)	1.06 (1.04, 1.08)
rs1878406	4	148393664	*EDNRA*	T	0.14	1.06 (1.04, 1.09)	1.04 (0.96, 1.12)	1.07 (1.04, 1.10)
rs17087335	4	57838583	*REST-NOA1*	T	0.18	1.06 (1.04, 1.09)	1.01 (0.94, 1.08)	1.05 (1.02, 1.07)
rs2505083	10	30335122	*KIAA1462*	C	0.43	1.06 (1.04, 1.08)	1.02 (0.97, 1.08)	1.04 (1.02, 1.06)
rs2048327	6	160863532	*SLC22A3-LPAL2-LPA*	C	0.4	1.06 (1.04, 1.08)	1.08 (1.02, 1.14)	1.07 (1.05, 1.10)
rs663129	18	57838401	*PMAIP1-MC4R*	A	0.25	1.06 (1.04, 1.08)	1.01 (0.95, 1.08)	1.02 (1.00, 1.05)
rs1561198	2	85809989	*VAMP5-VAMP8-GGCX*	T	0.46	1.06 (1.04, 1.08)	1.06 (1.01, 1.12)	1.05 (1.03, 1.07)
rs2047009	10	44539913	*CXCL12*	G	0.52	1.06 (1.04, 1.08)	1.03 (0.98, 1.09)	1.04 (1.02, 1.06)
rs10840293	11	9751196	*SWAP70*	A	0.56	1.06 (1.04, 1.08)	0.98 (0.93, 1.03)	1.05 (1.03, 1.07)
rs17464857	1	222762709	*MIA3*	T	0.85	1.06 (1.03, 1.09)	1.09 (1.01, 1.17)	1.06 (1.03, 1.09)
rs264	8	19813180	*LPL*	G	0.86	1.06 (1.03, 1.09)	1.12 (1.03, 1.21)	1.06 (1.03, 1.09)
rs273909	5	131667353	*SLC22A4-SLC22A5*	G	0.12	1.06 (1.03, 1.09)	1.02 (0.94, 1.10)	1.02 (0.99, 1.05)
rs2023938	7	19036775	*HDAC9*	C	0.1	1.06 (1.03, 1.09)	1.01 (0.93, 1.10)	1.07 (1.04, 1.11)
rs12190287	6	134214525	*TCF21**	C	0.63	1.06 (1.02, 1.10)	1.05 (0.99, 1.11)	1.06 (1.04, 1.08)
rs10953541	7	107244545	*7q22*	C	0.75	1.05 (1.03, 1.08)	1.02 (0.96, 1.08)	1.02 (0.99, 1.04)
rs4773144	13	110960712	*COL4A1/A2*	G	0.44	1.05 (1.03, 1.08)	1.01 (0.96, 1.06)	1.03 (1.01, 1.05)
rs10947789	6	39174922	*KCNK5*	T	0.77	1.05 (1.03, 1.08)	0.99 (0.93, 1.05)	1.03 (1.00, 1.05)
rs964184	11	116648917	*ZNF259-APOA5-APOA1*	G	0.14	1.05 (1.03, 1.08)	0.99 (0.92, 1.07)	1.05 (1.02, 1.08)
rs17514846	15	91416550	*FURIN-FES*	A	0.47	1.05 (1.03, 1.07)	1.07 (1.01, 1.13)	1.08 (1.06, 1.10)
rs4845625	1	154422067	*IL6R*	T	0.42	1.05 (1.03, 1.07)	1.02 (0.96, 1.07)	1.03 (1.01, 1.05)

rs6544713	2	44073881	*ABCG5-ABCG8*	T	0.32	1.05 (1.03, 1.07)	1.02 (0.97, 1.08)	1.05 (1.03, 1.07)
rs216172	17	2126504	*SMG6*	C	0.36	1.05 (1.03, 1.07)	1.01 (0.96, 1.07)	1.03 (1.01, 1.05)
rs2954029	8	126490972	*TRIB1*	A	0.54	1.04 (1.03, 1.06)	1.02 (0.97, 1.08)	1.05 (1.03, 1.07)
rs11203042	10	90989109	*LIPA*	T	0.45	1.04 (1.02, 1.06)	1.03 (0.97, 1.08)	1.03 (1.01, 1.05)
rs9319428	13	28973621	*FLT1*	A	0.3	1.04 (1.02, 1.06)	1.04 (0.98, 1.10)	1.02 (1.00, 1.04)
rs7136259	12	90081188	*ATP2B1*	T	0.42	1.04 (1.02, 1.06)	1.00 (0.95, 1.06)	1.01 (0.99, 1.03)
rs46522	17	46988597	*UBE2Z*	T	0.54	1.04 (1.02, 1.06)	1.04 (0.99, 1.10)	1.02 (1.00, 1.04)
rs2895811	14	100133942	*HHIPL1*	C	0.42	1.04 (1.02, 1.06)	1.02 (0.97, 1.08)	1.03 (1.01, 1.05)
rs4252120	6	161143608	*PLG*	T	0.71	1.03 (1.01, 1.06)	1.07 (1.01, 1.14)	1.05 (1.03, 1.07)
rs2252641	2	145801461	*ZEB2-ACO74093.1*	C	0.45	1.03 (1.01, 1.05)	1.01 (0.96, 1.06)	1.03 (1.01, 1.05)
rs12936587	17	17543722	*RAI1-PEMT-RASD1*	G	0.53	1.03 (1.01, 1.05)	1.08 (1.02, 1.13)	1.03 (1.01, 1.05)
rs17609940	6	35034800	*ANKS1A*	G	0.78	1.03 (1.00, 1.05)	1.06 (0.99, 1.13)	1.02 (1.00, 1.05)
rs6903956	6	11774583	*ADTRP-C6orf105*	A	0.38	1.00 (0.98, 1.02)	0.98 (0.93, 1.03)	1.00 (0.98, 1.02)

ESM Table 4. Sensitivity analysis of top findings using alternate diabetes and CAD definitions.

SNP	DM classification	outcome	N cases	N controls	OR (95% CI)	P
rs74617384	Probable and Possible Type 1 and 2	CAD incl. angina	3,968	11,698	1.38 (1.26, 1.51)	$3.2x10^{-12}$
		Excluding angina	3,293	11,698	1.41 (1.28, 1.56)	$4.2x10^{-12}$
	Probable Type 1 and 2	CAD incl. angina	3,258	10,303	1.39 (1.26, 1.53)	$8.3x10^{-11}$
		Excluding angina	2,671	10,303	1.43 (1.28, 1.59)	$6.3 x10^{-11}$
	Probable Type 2	CAD incl. angina	3,053	9,503	1.38 (1.25, 1.53)	$6.7x10^{-10}$
		Excluding angina	2,489	9,503	1.42 (1.27, 1.59)	$4.3x10^{-10}$
rs10811652	Probable and Possible Type 1 and 2	CAD incl. angina	3,968	11,698	1.19 (1.13, 1.26)	$6.0x10^{-11}$
		Excluding angina	3,293	11,698	1.23 (1.16, 1.30)	$2.0x10^{-12}$
	Probable Type 1 and 2	CAD incl. angina	3,258	10,303	1.21 (1.14, 1.28)	$2.2x10^{-10}$
		Excluding angina	2,671	10,303	1.24 (1.17, 1.32)	$1.5x10^{-11}$
	Probable Type 2	CAD incl. angina	3,053	9,503	1.20 (1.13, 1.27)	$4.6x10^{-9}$
		Excluding angina	2,489	9,503	1.24 (1.16, 1.32)	$2.1x10^{-10}$

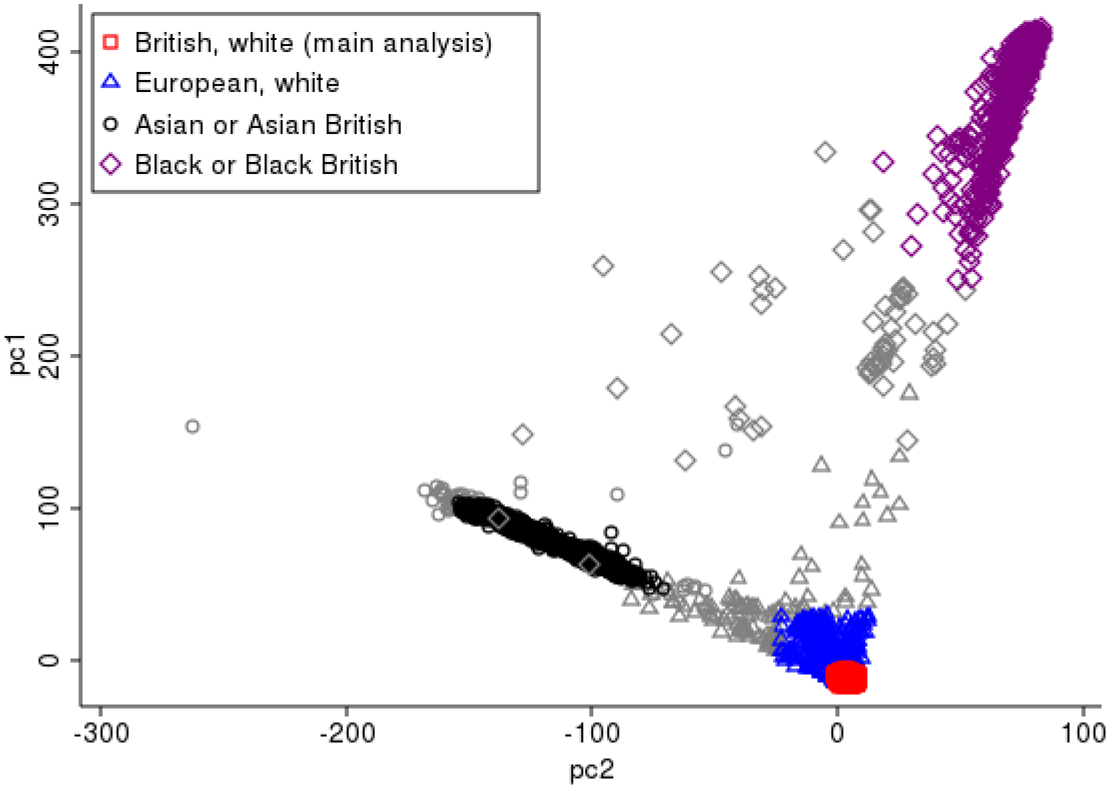

ESM Figure 1. Scatterplot of the first two components from a principal component analysis of individuals with diabetes from the UK Biobank, where individuals of self-reported white European background are shown with triangles, “Asian or Asian British” in circles and “Black or Black British” with diamond symbols. Excluded individuals are shown in grey.

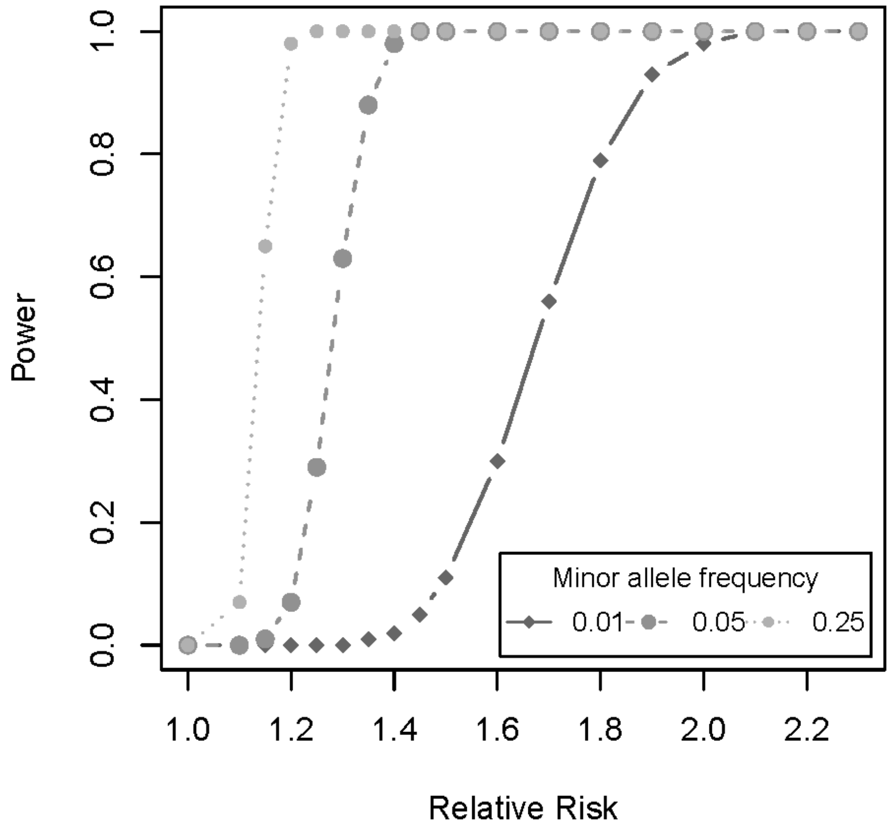

ESM Figure 2. Estimated power using the Genetic Association Study Power Calculator derived from the CaTS power calculator[5] to detect genetic variants at various relative effect sizes per allele, and minor allele frequencies, in our study of 3,968 CAD cases and 11,698 controls assuming an additive model of inheritance and a genome-wide significance threshold of $p<5x10^{-8}$.

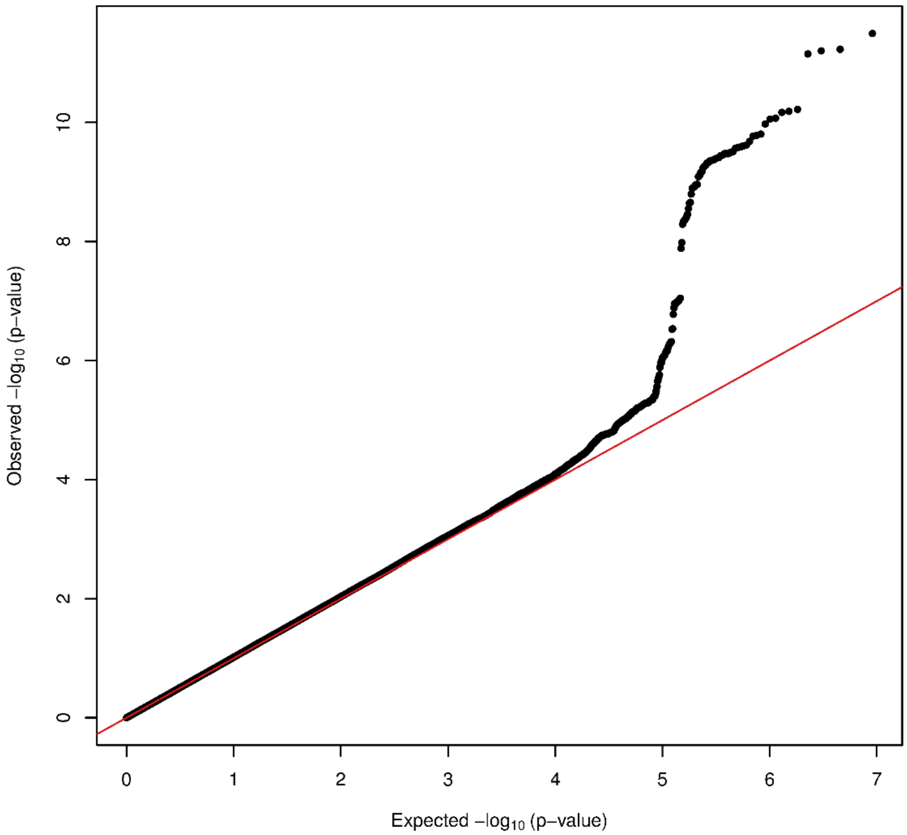

ESM Figure 3. Quantile-quantile plot illustrating the distribution of p-values across the genome compared to the expected distribution for our genome-wide association study of CAD (3,968 CAD cases and 11,698 controls) in participants with diabetes in UK Biobank. No indication of a systematic genomic inflation was observed (λ=1.017).

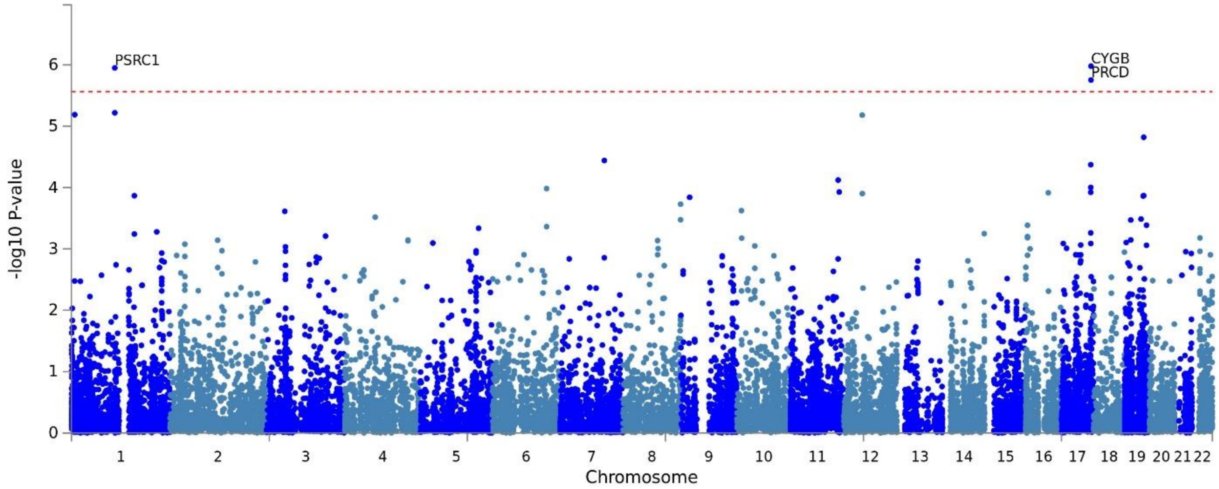

ESM Figure 4. Results from a gene-based analysis (MAGMA) suggesting association of *PSRC1*, *CYGB* and *PRCD* genes with CAD in patients with diabetes. Each tested gene is visualized as a dot with location on the genome on the x-axis and –log10-transformed p-values on the y-axis. Genome-wide significance (red dashed line in the plot) was defined at *P* = 0.05/18,205 (number of tested genes) = 2.746e-6.

References

1. Bycroft C, Freeman C, Petkova D, et al. Genome-wide genetic data on ~500,000 UK Biobank participants. *bioRxiv.* 2017.
2. de Leeuw CA, Mooij JM, Heskes T, Posthuma D. MAGMA: generalized gene-set analysis of GWAS data. *PLoS Comput Biol.* 2015;11(4):e1004219.
3. Watanabe K, Taskesen E, van Bochoven A, Posthuma D. Functional mapping and annotation of genetic associations with FUMA. *Nat Commun.* 2017;8(1):1826.
4. Subramanian A, Tamayo P, Mootha VK, et al. Gene set enrichment analysis: a knowledge-based approach for interpreting genome-wide expression profiles. *Proc Natl Acad Sci U S A.* 2005;102(43):15545-15550.
5. Skol AD, Scott LJ, Abecasis GR, Boehnke M. Joint analysis is more efficient than replication-based analysis for two-stage genome-wide association studies. *Nature genetics.* 2006;38(2):209-213.
